# Independent and Web-Based Advice for Infertile Patients Using Fertility Consult: Pilot Study

**DOI:** 10.2196/13916

**Published:** 2019-06-04

**Authors:** Aleida Gerarda Huppelschoten, Jan Peter de Bruin, Jan AM Kremer

**Affiliations:** 1 Department of Obstetrics and Gynaecology Radboud University Medical Center Nijmegen Netherlands; 2 Department of Obstetrics and Gynaecology Jeroen Bosch Hospital 's-Hertogenbosch Netherlands; 3 Scientific Institute for Quality in Healthcare Radboud University Medical Center Nijmegen Netherlands

**Keywords:** patient-centered care, remote consultation, decision making, telemedicine

## Abstract

**Background:**

Patient-centered care—that is, care tailored to personal wishes and needs of patients—has become increasingly important. It is especially relevant in health care areas where patients suffer from a high burden of disease, such as fertility care. At present, both diagnosis and treatment for infertile couples is provided at a single hospital. As a consequence, patients are not likely to receive optimal, independent advice regarding their fertility problems. Internet-based, independent advice could be feasible for large groups of patients because it is not limited by travel distance and overhead costs.

**Objective:**

The aim of this study was to explore the experiences of both patients and professionals with an online platform using video consultations for patients with infertility seeking independent advice for their fertility problem.

**Methods:**

This pilot study evaluated an online platform, Fertility Consult, where patients with infertility can get independent advice by a gynecologist through a video consultation, thus eliminating the need of meeting the doctor physically. Semistructured interviews were performed with 2 gynecologists and the chairman of the Dutch patients association. This information was used for a patients’ questionnaire about their first experiences with Fertility Consult, including questions about the level of patient-centeredness and shared decision making, using the Patient-Centered Questionnaire-Infertility (PCQ-Infertility) and the CollaboRATE questionnaire, respectively.

**Results:**

Of the first 27 patients enrolled at Fertility Consult, 22 responded (82%). Most patients (82%) visited Fertility Consult for a second opinion, seeking more personal attention and independent advice. The mean level of patient-centeredness on the PCQ-Infertility questionnaire was 2.78 (SD 0.58) on a scale of 0 to 3. For the CollaboRATE questionnaire (scale 0-9), patients provided a median score of 8.0 (range 7-9) on all 3 questions about shared decision making.

**Conclusions:**

Patients were satisfied with independent, well-prepared, Web-based advice; health care professionals felt they were able to provide patients with proper advice in a manner befitting patients’ needs, without any loss of quality. Future studies should focus more on the separation of advice and treatment and on Web-based consultations compared with face-to-face consultations to ascertain the possibility of increased patient involvement in the process to improve the level of patient-centered care.

## Introduction

In consulting rooms, health care professionals strive for a good relationship with their patients and want to provide them with the most optimal, person-centered advice and corresponding treatment. They are aware that more patient-centered care, which is focused on individual patients’ wishes and needs, will increase patients’ quality of life, improve health care professionals’ satisfaction in their daily work, and lower the dropout rates [1-5]. However, previous studies have shown that this level of patient-centeredness has not yet been achieved. Especially in health care areas where patients suffer from a high physical and psychological burden, such as fertility care, improvement is needed. These patients are in need of more emotional support and health care professionals who will listen to them attentively, and they want to be more involved in the decision-making process [6,7]. In daily clinical care, it might be difficult to fulfill all the above-mentioned requirements because of, for example, time pressure and the somewhat impersonal setting of a hospital.

Several improvement studies have already been conducted to overcome these problems with varying results [8-11]. Thus far, these strategies have mainly focused at optimizing existing concepts in hospital care. However, there is a need to look from a different perspective for a greater improvement in patient-centered care. Similar to several health care areas, fertility care can be considered as existing of 2 separate entities: (1) the diagnosis and advice phase and (2) the treatment phase. Currently, the same organization or hospital provides both advice and treatment. Consequently, doctors may have conflicting interests, as the revenues of advice and treatment are tied within the business model of their hospital organization. Is it then always possible to be completely objective and focused on the patient’s interests? In addition, patients’ experience can be affected by their impression that the advice is not entirely objective. Other problems that can arise in our current clinical organizations are limited time for shared decision making, loss of continuity of care, excessive treatment, large overhead costs in hospital settings, and consequently less patient satisfaction.

Organizing advice independently, described in the study by Clayton Christensen as a *solution shop* [12], may solve a large part of these problems. Patients who are looking for the most optimal treatment for their unfulfilled wish for a child could benefit from optimal independent advice by a senior expert in the field. Nowadays, the internet offers an optimal opportunity to make such independent advice feasible for larger groups of patients without the limitations of long-distance travel and with low overhead costs.

Therefore, the main aim of this pilot study was to explore the experiences of both infertile patients and professionals with using an online platform, which involved video consulting for patients with infertility seeking independent advice about their current fertility problem, thereby eliminating the need to meet the doctor physically. Meanwhile, this study will explore the possibilities of introducing video consulting in fertility care, including its advantages and disadvantages.

## Methods

### Study Design and Participants

In this clinical pilot study, both qualitative and quantitative methods were used to evaluate Fertility Consult as an independent platform to provide advice for infertile patients with the use of video consulting. In the qualitative part, professionals were asked about their experiences with Fertility Consult using semistructured interviews. The results of these interviews were used as input for a patients’ questionnaire—that is, the quantitative part of the study. To have more in-depth information about the level of shared decision making and patient-centered care, a modified version of the validated Patient-Centered Questionnaire-Infertility (PCQ-Infertility) and the validated CollaboRATE questionnaires were added to the questionnaire. Patients who were included in the pilot phase of Fertility Consult between February and April 2017 were asked to participate in the study.

### About Fertility Consult

Fertility Consult was developed in 2016 by 2 Dutch gynecologists who had a special interest in patient-centered innovations. Fertility Consult is an independent and secured online platform that patients can use to get advice or a second opinion on their current fertility problem. The online platform was created following the Dutch quality standard “NEN 7510” for information security in health care. The target population for the study included Dutch patients living abroad and patients receiving fertility treatment in the Netherlands and who considered continuing their fertility treatment abroad. The reason behind this limitation was to avoid any negative impact to collegial relationships in this early pilot phase of this service. We excluded patients who received fertility treatment in our own hospital regions. Patients could join the online platform through the Dutch fertility patient’s association “Freya.”

**Figure 1:**
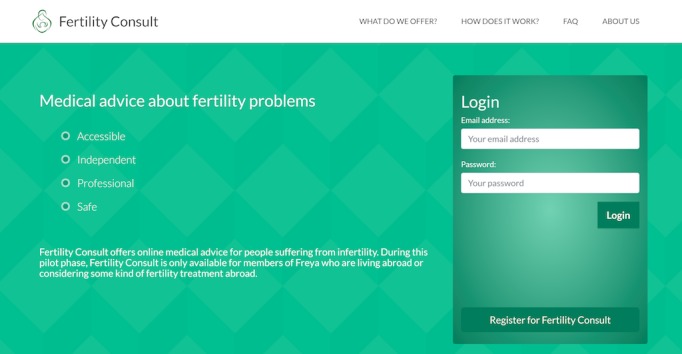
Homepage of Fertility Consult.

For the gynecologists to be able to provide well-informed advice, patients had to upload their medical data and complete questionnaires about their history and previous fertility trajectory—that is, the results of previous fertility testing and the details and outcomes of previous fertility treatments, if any (see Multimedia Appendix 1). After this process, they were able to schedule a video consultation (Skype) with 1 of the gynecologists. In case medical information was not sufficient for the health care professionals to give proper medical advice, patients were asked for additional information. After the video consultation, patients received a summary of the conversation and personal, independent advice in their personal environment of the website. Figure 1 shows a screenshot of the home page of Fertility Consult.

### Qualitative Part—The Interviews

For this part of the study, professionals were interviewed using semistructured interviews with a prospectively composed topic list. The professionals consisted of the 2 gynecologists performing the video consulting at Fertility Consult. In addition, the chairman of the Dutch fertility patients’ association “Freya” was interviewed to include her opinion on this new online platform as well.

This list consisted of the 7 domains of patient-centered fertility care (ie, accessibility of care, information provision, communication, respect for patients’ values, continuity of care, patient involvement, and professionals’ competence) [11], complemented by questions about the technical part of Fertility Consult and its future perspectives. By using this design, we offered the professionals flexibility in discussing all experiences that were of importance to them.

### Quantitative Part—The Questionnaires

For the quantitative part of the study, first, all patients who were eligible for participation were selected. Several patients subscribed for Fertility Consult once but never uploaded their personal information or requested for a video consult. Because of limited information being available about these patients, it was not possible to approach them for participation in the study. Therefore, only those patients were contacted who actually uploaded their medical file and had a video consultation with 1 of the 2 gynecologists.

The questionnaire consisted of questions about patients’ background characteristics and questions that were derived from the semistructured interviews. To verify the content of the questionnaires, a short interview was performed with 5 randomly chosen patients, after which the questionnaire was adjusted on minor details accordingly.

To gather more in-depth information about the level of patient-centeredness and shared decision making patients experienced at Fertility Consult, we extended our questionnaire with questions of the *PCQ-Infertility* and the *CollaboRATE questionnaire.* The first questionnaire is a validated instrument assessing the level of patient-centeredness in fertility care by measuring the specific experiences of patients [13]. The original questionnaire consists of 46 questions covering 7 different subscales (eg, information provision, communication, or respect for patients’ values). A higher score on the total PCQ-Infertility scale or 1 of its subscales (range 0-3) implies a higher level of patient-centeredness [13]. In our study, we used a modified version of the questionnaire as not all questions were suitable for our specific setting at Fertility Consult (eg, questions about the involvement of nurses in the fertility treatment). In total, we included 18 questions of the PCQ-Infertility covering 5 subscales.

The CollaboRATE questionnaire is a validated patient-reported measure of shared decision making, consisting of 3 questions following a clinical consult—that is, (1) “How much effort was made to help you understand your health issues?” (2) “How much effort was made to listen to the things that matter most to you about your health issues?” and (3) “How much effort was made to include what matters most to you in choosing what to do next?.” All questions were scored on a 9-point Likert scale (0=no effort was made and 9=every effort was made) [14]. All patients were invited by email to complete the online questionnaire. Nonresponders were sent 3 reminders.

### Data Analysis

All semistructured interviews were analyzed using a coding procedure in Atlas.ti (version 8, ATLAS.ti Scientific Software Development GmbH) to get an overview of the most important determinants. For the questionnaires, background characteristics and questions derived from the interviews were analyzed using descriptive statistics. Furthermore, the mean PCQ-Infertility total and subscale scores as well as the median CollaboRATE scores were calculated. All results were analyzed using the SPSS, version 22.

## Results

### Qualitative Part—The Interviews

Both gynecologists did not find the video consults to be different from a regular face-to-face consultation at the clinic. Appointments could be scheduled quite fast, and patients seemed comfortable in their home environment, making it a very accessible situation. Especially because it is the first time that patient and gynecologist see each other, the nonverbal communication of a video consultation is considered of additional value when comparing it with a consultation by telephone. No major technical problems occurred during the video consultations, and both gynecologists felt they could properly prepare for the consults on account of having all the uploaded medical information from their patients. Both gynecologists felt comfortable by solely focusing on giving patients proper advice, without other interests to consider. A limitation is that Fertility Consult in this setting could not be performed by less experienced doctors or residents, as patients obviously want to speak to an “authority” in the field of fertility care to seek the best advice. In the Netherlands, in vitro fertilization and intracytoplasmic sperm injection treatments are mainly provided by larger public and academic hospitals where residents and younger less experienced doctors work as well. Consequently, as patient numbers would increase at the platform, an optimal combination of younger and more experienced support staff and a cost-effective business model are necessary to keep this platform working.

The chairman of “Freya” was very satisfied with an initiative such as Fertility Consult and found it especially important for patients with infertility who do not feel heard by their own gynecologist or for patients who want to go abroad but do not know exactly if and how this can actually increase their chances of getting pregnant. According to her, a point of interest should be how to reach low-literate patients.

### Quantitative Part—The Questionnaires

The pilot group comprised 27 patients. Of these, 22 responded (response rate 81.5%). The median age was 37 years (range 29-48), and 16.7% (4/27) lived abroad. The characteristics of patients are summarized in Table 1.

After patients uploaded their medical information at Fertility Consult and asked for a consultation, the median waiting time before patients actually had a consultation was 5 days (range 2-28 days). The results of the second part of the questionnaire are summarized in Table 2.

**Table 1:** Background characteristics (N=22).

Characteristics		Values
**Level of education, n (%)**		
	High^a^	91
	Other	9
Living abroad, n (%)		17
**Ethnic background, n (%)**		
	White	86
	Other	14
**Previous treatment, n (%)**		
	None	27
	Non-ART^b,c^	9
	ART^d^	64
**Duration of infertility (years)**		
	<2	21
	2-5	42
	>5	37
Pregnant, %		16

^a^High level of education=higher professional education or university.

^b^ART: assisted reproductive technology.

^c^Included ovulation induction and intrauterine insemination with or without controlled ovarian stimulation.

^d^Encompassed in vitro fertilization intramuscular, intracytoplasmic sperm injection, cryopreservation, and testicular sperm extraction.

**Table 2:** In-depth questions per patient group (N=22).

Characteristics		Values
**Reason visiting FC^**a**^, n (%)**		
	First opinion	18
	Second opinion	82
**How did you know about FC, n (%)**		
	Patient organization	82
	Family	9
	Google	9
**Clarity website FC, n (%)**		
	Not at all	0
	Little	0
	Mostly	46
	Absolutely	54
**Comprehensibility questionnaire, n (%)**		
	Not at all	0
	Little	0
	Mostly	50
	Absolutely	50
**Time completing questionnaire (min)**		
	Median (range)	30 (10-60)
**Satisfaction creating own medical file, n (%)**		
	Not at all	0
	Little	10
	Absolutely	90
**Contact with other patients through FC, n (%)**		
	Yes	18
	No	82
**Recommend FC to others, n (%)**		
	Yes	90
	Maybe	10
	No	0
Total score for FC (0-10), median (range)		9 (7-10)
**Willing to pay for FC**		
	Yes, n (%)	86
	No, n (%)	14
	Median (range), €	€60 (€10-250)

^a^FC: Fertility Consult.

Most importantly, 82% of patients visited Fertility Consult for a second opinion; they searched for more personal attention than they got at their own hospital and independent advice. Patients who wanted to go abroad were looking for advice on which hospital or doctor to go to. A total of 90.5% of patients would definitely recommend Fertility Consult to their family or friends. The median overall rate for Fertility Consult was 9 (range 7-10) at a scale of 1 to 10. Furthermore, 86% of patients were willing to pay a median amount of €60 for independent advice at Fertility Consult (range €10-250).

**Table 3:** Results of the modified Patient-Centered Questionnaire-Infertility and CollaboRATE questionnaire (N=22).

Characteristics			Values
**Patient-Centered Questionnaire-Infertility, range 0-3 (mean, SD)**			
	**Total score**		2.78 (0.58)
		Communication	2.88 (0.51)
		Patients involvement	2.81 (0.50)
		Respect for patients’ values	2.81 (0.55)
		Staff’s competence	2.77 (0.55)
		Organization of care	2.57 (0.74)
**CollaboRATE, range 0-9 (median, range)**			
	Helping to understand health issues		8 (7-9)
	Listen to things that matter most		8 (7-9)
	What to do next		8 (7-9)

The results of the PCQ-Infertility and CollaboRATE questionnaires are summarized in Table 3. The mean total PCQ-Infertility score was 2.78 (SD 0.58) on a range of 0 to 3. The highest rating was provided to the subscale “Communication” (mean 2.80, SD 0.51) and the lowest rating for “Organization of care” (mean 2.57, SD 0.74).

For the CollaboRATE questionnaire, patients provided a median score of 8.0 (range 7-9) on a scale of 1 to 9 on all 3 questions about shared decision making.

## Discussion

### Principal Findings

This is the first pilot study exploring both patients’ and professionals’ experiences with an online platform using video consulting for infertile patients seeking independent advice about their fertility problem. In general, it was found that patients were satisfied with independent, well-prepared Web-based advice. Professionals felt they can provide patients with proper advice that caters to and fulfills patients’ needs, without any loss of quality. As this was a small pilot study, caution should be exercised with respect to conclusions before the results can be generalized to larger groups or other health care areas.

Fertility Consult was developed to provide infertile patients with advice independently of other interests or benefits. This is not too common in health care yet but nonetheless, an interesting topic. Already about 2 decades ago, Christensen et al wrote that health care might be the most change-averse industry [12]. He found a resistance to several low-cost alternatives, which was not in the best interest of the patients. Doctors sometimes have difficulty with terms such as market force and innovations. A division between diagnosis and advice on the one hand and providing treatment on the other hand might be one of these difficult subjects as well. With this pilot study, it was shown that such independent advice is possible for a specific patient population in fertility care, with positive experiences of both participating patients and professionals. More research about this subject is, however, needed to provide more insight in the advantages and disadvantages of implementing the introduction of an independent treatment advice for patients in daily clinical care.

In this study, we provided independent advice in an online setting using video consulting. By making use of the internet, we expected to gain different advantages compared with a face-to-face consultation. It is, for example, an optimal tool to reach many patients from different areas in the Netherlands and abroad. Moreover, a Web-based discussion with a doctor in a neutral and safe environment for the patient (ie, at home or at work) prevents long-distance travel and immediately provides the appeal of an “independent counseling clinic” without the focus on therapy.

### Comparison With the Literature

The literature on this subject is rather scarce, but some studies found that video consulting could account for different shortcomings in health care. In the Netherlands, the study by Schers et al showed that elderly patients in general practitioner (GP) practices were in particular skeptical to its use, and technical failures were mentioned as an important pitfall [15]. On the other hand, younger patients and patients who can handle computers might see benefits from video consulting. As patients with infertility are relatively young with a high demand of involvement in the decision-making process, fertility care could be a suitable field for video consulting. In other countries, especially those where patients have to travel long distances for access to specialist care, video consultation might provide a possibility to provide more medical services for these patients. In Australia, more than 100 GPs were asked to review video vignettes covering different patient scenarios. A total of 72% to 100% of the GPs agreed on the differential diagnoses of the scenarios, and GPs in larger practices especially were more positive toward video consulting [16]. In Sweden, similar results were found as GPs found video consultation an opportunity to provide education and ability for their patients to ask questions [17]. Furthermore, the safety of video consulting was studied, finding that good communication was essential for patients’ perception of security during the consultation [18]. A study of Westra et al about the use of video consulting in plastic surgery found that patients who received a video consultation 6 weeks after their surgery had a higher general satisfaction and less waiting time than patients with traditional in-person consultation. However, patients receiving Web-based consultation were less satisfied with the patient-physician communication [19].

Our study showed that the level of communication satisfied both patients and professionals. On the PCQ-Infertility questionnaire, the subscale communication even received the highest ratings. However, it remains interesting whether patients are really comfortable talking to the doctor through a computer or is it just a face-to-face contact they prefer as “the golden standard.” On the basis of the literature, it seems like the latter is true, but video consulting could definitely overcome problems such as travel distance, waiting times, and impersonal hospital settings without a significant loss of quality of the consultation. This is especially important for patients with infertility, as not all kinds of fertility treatment are provided in all Dutch hospitals and require patients to travel large distances. Moreover, patients often suffer from psychological burden because of infertility and could, therefore, benefit from a doctor really listening to them in the safe and well-known environment of their own house.

Topics of safety and quality are obviously important when implementing an initiative such as Fertility Consult. As telemedicine is an upcoming way of providing health care, many national and international standards for video consulting and corresponding initiatives are already developed. For example, the American Telemedicine Association provides guidelines for managing patient safety [20]. Fertility Consult was developed following the Dutch quality standard “NEN 7510” for information security in health care, which is derived from the international ISO 27001 standard. Therefore, no safety and quality issues should be expected with the use of Fertility Consult.

### Strengths and Weaknesses

The use of an online and secure platform and video consulting to provide independent advice for patients with infertility has never been studied before and is, therefore, one of the strengths of this study. As we used both qualitative and quantitative techniques, we were able to put the results of our questionnaires in a broader perspective and considered the opinions of patients, participating gynecologists, and the Dutch fertility patient association. Subsequently, we used 2 validated questionnaires to provide more information about the level of patient-centeredness and shared decision making patients experienced during their consultation. It is already known that patients in fertility care need more patient-centric care and shared decision making, so it is interesting that our pilot study seeks for a relationship with video consultation and the possibility of providing patients with independent advice.

Some limitations should be mentioned as well. First, because of the pilot setting, we used a small sample size. Although the response rate was quite high (81.5%), it would had been interesting as well to include patients who registered for Fertility Consult once but never uploaded their information and asked for a Web-based consultation. This might be a patient group without a strong need for a second opinion or independent advice. However, technical struggles for making an appointment or other difficulties in the process cannot definitely be ruled out, as we had no contact information to approach these patients for participation.

Furthermore, we used a modified version of the PCQ-Infertility questionnaire, as not all questions were applicable to the setting of video consultation. As the questionnaire is not validated and uses only some questions, we cannot compare our results with other studies using the PCQ-Infertility. However, by using these questions, we could provide a general overview of the degree of patient-centeredness that patients experienced when participating in Fertility Consult.

Finally, only the women of the infertile couple completed the questionnaires. This might have caused bias, although the literature shows that both women and their partners have comparable experiences with fertility care [21].

### Conclusions

In conclusion, this pilot study explored the experiences of both patients and professionals with the online platform Fertility Consult providing patients with an independent advice about their fertility problem. This study shows a good satisfaction rate and a high level of patient-centeredness and shared decision making of patients who had a Web-based video consult. Patients appreciate the personal attention they received and independent advice. Professionals had positive experiences with the online platform as well, but they mentioned several areas for improvement. For them, the platform preferably should improve in terms of a more sophisticated and intuitive (mobile) interface for both patients and professionals. The process of scheduling appointments was bothersome, and it would further empower patients if they were able to schedule the appointment themselves. The platform should support the professional in generating written advice based on the answers from the patient questionnaires. Furthermore, it should be easier to track the flow of the patient population and to generate aggregated data for the population.

As this study showed positive experiences of patients and professionals with Fertility Consult, a next step would be to implement this pilot. We could, therefore, think of expanding the design of the website and adding an interactive mobile app with algorithms for evidence-based advice and coaching. As a next step, it would be interesting to compare quality of care of the online network versus standard care, focusing on lifestyle improvement, chance of spontaneous pregnancy, birth of a healthy child, psychological well-being, and patient-reported outcomes and experiences. Future studies should also focus on the interesting topic of the separation of medical advice and treatment and on Web-based consultations compared with face-to-face consultations to ascertain if patient involvement in the process can be further increased to improve the level of patient-centered care.

## Appendix

Multimedia Appendix 1 Fertility Consult questionnaires.

## Abbreviations

FC: Fertility Consult
GP: general practitioner
PCQ-Infertility: Patient-Centered Questionnaire-Infertility
