# Supplementary material for: Independent and Web-Based Advice for Infertile Patients Using Fertility Consult: Pilot Study
Source: JMIR Form Res. 2019 Jun 4;3(2):e13916. doi: 10.2196/13916 (PMC6746069; doi:10.2196/13916)
Supplement: Multimedia Appendix 1 [file formative_v3i2e13916_app1.docx]

**Questionnaire 1: Fertility questionnaire**

**1. General information**

1.1 Choose the situation that matches your situation

Child wish within a man-woman relationship

Child wish within a woman-woman relationship

Child wish without a relationship (single)

1.2 First name woman: …

1.3 Surname woman: …

1.4 Date of birth woman: … (day / month / year)

1.5 First name partner: …

1.6 Surname partner: …

1.7 Date of birth partner: … (day / month / year)

1.8 Child wish since …. (day / month / year)

1.9 Have you ever been pregnant?

Yes

No (skip question 2)

1.10 Did your partner ever conceive a pregnancy?

Yes

No

**2. Information about previous pregnancies**

**First pregnancy**

2.1 Year of pregnancy: …

2.2 Was this a twin pregnancy?

Yes

No

2.3 How did you conceive?

Spontaneously

After fertility treatment

2.4 Mode of delivery

Spontaneously

Ceasarean section

Miscarriage

Ectopic pregnancy

2.5 Gestation in weeks: …

2.6 Gender

Boy

Girl

Two boys

Two girls

A boy and a girl

2.7 Birth weight in grams: …

2.8 Was this pregnancy within the current relationship?

Yes

No

**Second pregnancy**

2.9 Year of pregnancy: …

2.10 Was this a twin pregnancy?

Yes

No

2.11 How did you conceive?

Spontaneously

After fertility treatment

2.12 Mode of delivery

Spontaneously

Caesarean section

Miscarriage

Ectopic pregnancy

2.13 Gestation in weeks: …

2.14 Gender

Boy

Girl

Two boys

Two girls

A boy and a girl

2.15 Birth weight in grams: …

2.16 Was this pregnancy within the current relationship?

Yes

No

**Third pregnancy**

2.17 Year of pregnancy: …

2.18 Was this a twin pregnancy?

Yes

No

2.19 How did you conceive?

Spontaneously

After fertility treatment

2.20 Mode of delivery

Spontaneously

Caesarean section

Miscarriage

Ectopic pregnancy

2.21 Gestation in weeks: …

2.22 Gender

Boy

Girl

Two boys

Two girls

A boy and a girl

2.23 Birth weight in grams: …

2.24 Was this pregnancy within the current relationship?

Yes

No

**Fourth pregnancy**

2.25 Year of pregnancy: …

2.26 Was this a twin pregnancy?

Yes

No

2.27 How did you conceive?

Spontaneously

After fertility treatment

2.28 Mode of delivery

Spontaneously

Caesarean section

Miscarriage

Ectopic pregnancy

2.29 Gestation in weeks: …

2.30 Gender

Boy

Girl

Two boys

Two girls

A boy and a girl

2.31 Birth weight in grams: …

2.32 Was this pregnancy within the current relationship?

Yes

No

**3. History of the woman**

3.1 Did you ever undergo surgery?

Yes

No (go to 3.2)

3.1.1 When en for what reason did you undergo surgery? …

3.2 Did you ever had an appendicitis?

Yes

No (go to 3.3)

3.2.1 Was the appendicitis complicated / ruptured?

Yes

No

3.3 Did you ever have an infection at your ovaries?

Yes

No

3.4 Do or did you have a chronic condition?

Yes

No (go to 3.5)

3.4.1 What kind of condition did you have and when was this? …

3.5 Did you ever have an IUD (intra-uterine device)?

Yes

No

3.6 Further comments about your general health: …

**4. Family history of the woman**

4.1 Age of menopause of your mother: …

4.2 Are there any fertility problems in your family?

Yes

No

4.3 Are there any congenital abnormalities in your family?

Yes

No (go to 4.4)

4.3.1 What kind of congenital abnormalities?

4.4 Are multiple miscarriages (more than 2) in your family?

Yes

No

4.5 Did anybody in your family have a thrombosis or embolus?

Yes

No

4.6 Are there any hereditary illnesses in your family?

Yes

No (go to 4.7)

4.6.1 What kind of illnesses?

4.7 Did anybody in your family have cancer?

Yes

No (go to 5)

4.7.1 What kind of cancer and with whom?

**5. General information of the woman**

5.1 Race

Caucasian

Negroid

Mediterranean

Asian

Hindustani

Other, namely …

5.2 Do you understand the Dutch language?

Yes

No, but I do understand ….

5.3 Highest education: …

5.4 What is your profession? …

5.5 Do you have an occupation currently?

Yes, for … hours a week

No

5.6 Do you suffer from anxiety of depressive feelings?

Yes

No (go to 5.7)

5.6.1 Do you want to tell us more about these feelings? ...

5.7 Do you use any medication?

Yes

No (go to 5.8)

5.7.1 What kind of medication and in what dosage? …

5.8 Do you smoke?

Yes

No (go to 5.9)

5.8.1 How many cigarettes a day do you smoke? ...

5.9 Do you use any alcohol?

Yes

No (go to 5.10)

5.9.1 How many glasses of alcohol do you drink per week? …

5.10 Do you use any drugs?

Yes

No (go to 5.11)

5.10.1 What kind of drugs do you use and how often? …

5.11 Do you have any allergies?

Yes

No (go to 5.12)

5.11.1 What kind of allergies do you have? …

5.12 What is your length? …

5.13 What is your weight? …

**6. Specific information about the woman**

6.1 At what age was your first period? …

6.2 What is the date of your last period? … (day / month / year)

6.3 What is the length between the start of two following periods?

Mean: … (days)

Shortest cycle: … (days)

Longest cycle: … (days)

6.4 Which of the following is applicable to you?

Irregular cycle

Dysmenorrhea

Ever had an I.U.D.

Acne

Excessive body hair

Hair loss

Losing water from the nipples

Hot flushes

Change of weight (>5kg)

None of these

6.5 Did you ever have a sexual transmitted disease (STD)?

Yes

No (go to 7)

6.5.1 What kind of STD did you have? …

**7. History of the partner (male)**

7.1 Did you ever undergo surgery?

Yes

No (go to 3.2)

7.1.1 When en for what reason did you undergo surgery? …

7.2 Do or did you have a chronic condition?

Yes

No (go to 7.3)

7.2.1 What kind of condition did you have and when was this? …

7.3 Did you ever have an IUD (intra-uterine device)?

Yes

No

7.4 Further comments about your general health: …

**8. Family history of the partner (male)**

8.1 Are there any fertility problems in your family?

Yes

No

8.2 Are there any congenital abnormalities in your family?

Yes

No (go to 8.3)

8.2.1 What kind of congenital abnormalities?

8.3 Are multiple miscarriages (more than 2) in your family?

Yes

No

8.4 Did anybody in your family have a thrombosis or embolus?

Yes

No

8.5 Are there any hereditary illnesses in your family?

Yes

No (go to 8.6)

8.5.1 What kind of illnesses?

8.6 Did anybody in your family have cancer?

Yes

No (go to 9)

8.6.1 What kind of cancer and with whom?

**9. General information of the woman**

9.1 Race

Caucasian

Negroid

Mediterranean

Asian

Hindustani

Other, namely …

9.2 Highest education: …

9.3 What is your profession? …

9.4 Do you have an occupation currently?

Yes, for … hours a week

No

9.5 Do you suffer from anxiety of depressive feelings?

Yes

No (go to 9.6)

9.5.1 Do you want to tell us more about these feelings? ...

9.6 Do you use any medication?

Yes

No (go to 9.7)

9.6.1 What kind of medication and in what dosage? …

9.7 Do you smoke?

Yes

No (go to 9.8)

9.7.1 How many cigarettes a day do you smoke? …

9.10 Do you use any alcohol?

Yes

No (go to 9.11)

9.10.1 How many glasses of alcohol do you drink per week? …

9.11 Do you use any drugs?

Yes

No (go to 9.12)

9.11.1 What kind of drugs do you use and how often? …

9.12 What is your length? …

9.13 What is your weight? …

**10. Specific information about the partner (male)**

Did you suffer from any of these genital problems?

10.1 Testicle that did not descend

Yes

No

10.2 Trauma at the testicle

Yes

No

10.3 Torsi of the testis

Yes

No

10.4 Infection of the testis

Yes

No

10.5 Mumps as an adult

Yes

No

10.6 Seksually transmitted disease (STD)

Yes

No

10.6.1 What kind of STD did you have? …

10.7 Hernia inguinalis

Yes

No

10.8 Hydrocele

Yes
 No

Sexual history

10.9 Do you have a decreased libido?

Yes

No

10.10 Do you have any problems with your erection?

Yes

No

10.11 Do you have any problems with the discharge of seed?

Yes

No

**11. Seksual history**

11.1 How often do you have intercourse? … many times per week

11.2 Do you, madam, know when you are fertile?

Yes

No

11.3 Do you, madam, have pain during intercourse?

Yes

No

11.4 Did you have any negative sexual experiences?

Yes

No

**12. Remarks**

Do you have final remarks or questions that might be important?

………………………………………………………………………………………………………….

*Thank you for filling in the questionnaire. If you already had any fertility tests and / or treatment you can continue with questionnaire 2 and 3. If you have completed all questionnaires that are applicable you may now schedule your first video consultation.*

**Questionnaire 2: Previous diagnostic tests**

**1. Diagnostic tests**

1.1. Did you receive diagnostic tests?

Yes

No (go to end)

If yes, in which hospital?

2. Sperm tests

2.1 Has a sperm test been performed?

Yes

No (go to 3)

2.2 What did the doctor tell you about the sperm quality?

Good

Moderate

Poor

I don’t know

2.3 Do you have a copy of the result of the sperm count?

Yes

No (go to 3)

2.4 What is the result of the sperm count?

Volume:

Concentration:

Morfology:

Motility A (% fast moving sperm cells)

Motility B (% moderate moving sperm cells)

Motility C (% at one place moving sperm cells)

Motility D (% not moving sperm cells)

**3. Hormonal tests**

3.1 Did the doctors perform hormonal tests by drawing blood?

Yes

No (go to 4)

3.2 What where the results of these tests?

FSH

LH

AMH

TSH

Oestradiol

Progestron

Testosteron

Prolactine

**4. Ovulation**

4.1 Did your clinic check the quality of your ovulation?

Yes

No (go to 5)

4.2 Was your ovulation optimally?

Yes

No

I don’t know

4.3 How did they test the quality of the ovulation?

By my story (having a regular period)

By a temperature curve

By ultrasounds to follow my cycle

By blood tests

By urine tests

5. Post coitumtest

5.1 Did you undergo a post coitumtest?

Yes

No (go to 6)

5.2 Was the result positive (i.e. moving sperm cells were seen)?

Yes

No

**6. Chlamydia tests**

6.1 Did you undergo a test to check for Chlamydia

Yes

No (go to 7)

6.2 What was the result of this test?

Positive (have had Chlamydia)

Negative (never have had Chlamydia)

I don’t know

**7. Uterosalpingography**

7.1 Did you undergo an uterosalpingography?

Yes

No (go to 8)

7.2 What was the result of this test?

Normal

1 Fallopian tube was closed

Both Fallopian tubes were closed

Different shape of the uterus

I don’t know

**8. Endoscopic surgery (abdomen)**

8.1 Did you undergo endoscopic surgery at the abdomen?

Yes

No (go to 9)

8.2 What was the result of the surgery?

Normal

Abnormality at the womb

Abnormality at the right Fallopian tube

Abnormality at the left Fallopian tube

Abnormality at the right ovary

Abnormality at the left ovary

Endometriosis

Adhesions

I don’t know

**9. Endoscopic surgery (womb)**

9.1 Did you undergo endoscopic surgery at the womb?

Yes

No (go to part 3 of the questionnaire)

9.2 What was the result of the surgery?

Normal

Polyp

Fibroid

Adhesions

Different shape of the womb / septum

I don’t know

*Thank you for filling in the questionnaire. During your consultation with the fertility specialist your answers wil further be explored if necessary. If you have completed all three questionnaires you may now schedule your first video consultation.*

**Questionnaire 3: Previous Fertility Treatments**

1. **Previous treatments**

1.1 Did you receive fertility treatment?

Yes

No (go to end)

**2. Ovulation induction**

2.1 Did you receive fertility treatment for ovulation disorders?

Yes

No (go to 3.1)

2.2. Did you use hormone tablets for ovulation induction?

Yes

No (go to 2.6)

2.3 How many times (cycles) did you use hormone tablets?

…. (number)

2.4 What was the name of the hormone tablets?

Clomiphene citrate

Tamoxifen

Metformin

Other

2.5 In which clinic did these treatment cycles take place?

………..… (name)

2.6 Did you use hormone injections for ovulation induction?

Yes

No (go to 2.10)

2.7 How many times (cycles) did you use hormone injections?

…. (number)

2.8 What was the name of the hormones used?

Puregon

Gonal-F

Menopur

Fostimon

Other

Don’t know

2.9 In which clinic did these treatment cycles take place?

………..… (name)

2.10 Did you receive other treatments for ovulation induction?

Yes

No (go to 3.1)

2.11 What kind of other ovulation induction treatments did you receive?

GnRH pump

Laparoscopic/ laser electrocoagulation of the ovaries

Other

2.12 In which clinic did these treatment cycles take place?

………..… (name)

**3. IUI**

3.1 Did you receive treatments with intra uterine insemination (IUI)?

Yes

No (go to 4.1)

3.2 How many times (cycles) did you receive IUI?

…. (number)

3.3 Did you use hormonal stimulation during the IUI treatments?

Yes

No (go to 3.5)

3.4 What kind of hormonal stimulation did you use?

Hormone tablets

Hormone injections

First hormone tablets, followed by injections in later cyclus

Don’t know

3.5 Was donor sperm used?

Yes

No

3.6 What was the quality of the inseminated sperm in the IUI-cycles?

Good

Mediocre

Poor

Don’t know

3.7 In which clinic did these treatment cycles take place?

………..… (name)

**4. IVF/ ICSI**

4.1 Did you receive IVF- or ICSI-treatments?

Yes

No (go to 6.1)

*Would you please answer the following questions for each IVF- or ICSI-treatment cycle you received?*

Treatment cycle 1

4.2 Kind of treatment cycle

IVF

ICSI

4.3 In which clinic did these treatment cycle take place?

………..… (name)

4.4 Was the treatment cycle performed with your own germ cells or with the germ cells of a donor? Multiple answers are possible.

Own oocytes

Own sperm

Donor oocytes

Donor sperm

4.5 What was the starting dose of FSH in units/ day?

…. (number)

4.6. How many oocytes were harvested at the oocyte retrieval?

…. (number)

4.7 How many embryos developed?

…. (number)

4.8 How many embryos were transferred to your uterus?

…. (number)

4.9 What was the quality of the embryos?

Good

Mediocre

Poor

Don’t know

4.10 How many embryos were frozen?

…. (number)

4.11 What was the result of the treatment cycle?

Not pregnant

Singleton pregnancy

Twin pregnancy

Non vital pregnancy

Ectopic pregnancy

Repetition of same questions for treatment cycles 2 to 4

**5. Frozen embryos**

*Transfer of frozen embryos (cryo cycles)*

5.1 Have embryos of IVF or ICSI been frozen?

Yes

No (go to end)

*Would you please answer the following questions for each frozen embryo treatment cycle you received?*

5,2 How many frozen embryos were transferred tot the uterus?

|  | None | One | Two | Three or more |
| --- | --- | --- | --- | --- |
| Cryo cycle 1 |  |  |  |  |
| Cryo cycle 2 |  |  |  |  |
| Cryo cycle 3 |  |  |  |  |
| Cryo cycle 4 |  |  |  |  |
| Cryo cycle 5 |  |  |  |  |
| Cryo cycle 6 |  |  |  |  |
| Cryo cycle 7 |  |  |  |  |
| Cryo cycle 8 |  |  |  |  |
| Cryo cycle 9 |  |  |  |  |

5.3 What was the result of the cry treatment cycle?

Multiple answers are possible.

|  | Singleton pregnancy | Twin pregnancy | Non vital pregnancy | Ectopic pregnancy |
| --- | --- | --- | --- | --- |
| Cryo cycle 1 |  |  |  |  |
| Cryo cycle 2 |  |  |  |  |
| Cryo cycle 3 |  |  |  |  |
| Cryo cycle 4 |  |  |  |  |
| Cryo cycle 5 |  |  |  |  |
| Cryo cycle 6 |  |  |  |  |
| Cryo cycle 7 |  |  |  |  |
| Cryo cycle 8 |  |  |  |  |
| Cryo cycle 9 |  |  |  |  |

5.4 Are there still frozen embryos left?

Yes

No (go to 6.1)

5.4.1 How many frozen embryos are left?

…. (number)

**6. Other medical treatments**

6.1 Did you receive other medical treatments for infertility such as for example surgery?

Yes

No (go to 7.1)

6.2 What kind of other medical treatments did you receive?

………………………………………………………………………………………………………….

**7. Alternative therapies?**

7.1 Did you or your partner receive alternative therapies for infertility?

Yes

No (go to 8.1)

7.2 What kind of alternative therapies?

Multiple answers possible.

Homeopathy

Acupuncture

Herbal medicine

Other

**8. Remarks**

8.1 Do you have final remarks or questions on the fertility treatments you received?

………………………………………………………………………………………………………….

*Thank you for filling in the questionnaire. During your consultation with the fertility specialist your answers wil further be explored if necessary. If you have completed all three questionnaires you may now schedule your first video consultation.*
